# Intradomain Allosteric Regulation of Soluble Epoxide Hydrolase by Its Substrates

**DOI:** 10.3390/ijms252413496

**Published:** 2024-12-17

**Authors:** Shin Matsumura, Ayano Shida, Moeno Tsuchii, Mika Wada, Jimmy Charneau, Motonori Tsuji, Keiji Hasumi, Eriko Suzuki

**Affiliations:** 1 Department of Applied Biological Science, Tokyo University of Agriculture and Technology, Fuchu 183-8509, Japan; s232554z@st.go.tuat.ac.jp (S.M.); s235448q@st.go.tuat.ac.jp (A.S.); fy2228@go.tuat.ac.jp (M.T.); hasumi@cc.tuat.ac.jp (K.H.); 2 Department of Research and Development, TMS Co., Fuchu 183-0055, Japan; wadam@tms-japan.co.jp (M.W.); charneau@tms-japan.co.jp (J.C.); 3 Institute of Molecular Function, Saitama 341-0037, Japan; motonori@molfunction.com

**Keywords:** soluble epoxide hydrolase, phosphatase, enzyme kinetics, inflammation, lysophosphatidic acid

## Abstract

Soluble epoxide hydrolase (sEH) is a bifunctional enzyme with epoxide hydrolase activity in the C-terminal domain (C-EH) and lipid phosphate phosphatase activity in the N-terminal domain (N-phos). The C-EH hydrolyzes bioactive epoxy fatty acids such as epoxyeicosatrienoic acid (EET). The N-phos hydrolyzes lipid phosphomonesters, including the signaling molecules of lysophosphatidic acid (LPA). Here, we report that the C-EH and N-phos are reciprocally regulated by their respective substrates. Full-length sEH (sEH-FL) showed positive cooperativity toward the substrate for each domain. Similar cooperativity was found when truncated enzymes having only C- and N-terminal domains, sEH-C and sEH-N, respectively, were used, suggesting an intra-domain nature of the cooperativity. In addition, the N-phos substrate LPA inhibited C-EH activity in sEH-FL and sEH-C equally. Similarly, the C-EH substrate EET inhibited N-phos activity. Structural and kinetic data suggest the presence of allosteric sites in each domain of the sEH enzyme, which share the binding of LPA and EET. Thus, each of the two sEH activities is regulated by a substrate of its own and by that of the other domain. This mechanism may explain why sEH has evolved to have two different enzyme activities, which possibly allows sEH to balance the metabolism of bioactive lipids.

## 1. Introduction

Mammalian soluble epoxide hydrolase (sEH) has lipid epoxide hydrolase activity in its C-terminal domain (C-EH) and lipid phosphate phosphatase activity in its N-terminal domain (N-phos) [1,2,3]. The C-EH catalyzes the hydrolysis of epoxy fatty acids such as epoxyeicosatrienoic acids (EETs), which are potent endogenous signaling molecules implicated in anti-inflammation, vascular dilation, endothelial cell hyperpolarization, angiogenesis, neuroprotection, and analgesia (anti-hyperalgesia) [4,5]. The N-phos hydrolyzes lipid phosphates such as lysophosphatidic acid (LPA) and intermediates of the cholesterol biosynthesis [6,7,8]. sEH-deficient mice exhibit resistance to diverse inflammatory conditions [9,10,11,12,13]. Although this trait has been thought to be dependent on the C-EH function, our data suggest that the N-phos function plays a significant role in the suppression of vascular endothelial activation.

Protein functions are dynamically regulated by allostery, which enables conformational interaction between one domain and others. A structural change in one domain upon the inhibitor or substrate binding may affect the function of the other domain, which is the so-called interdomain allostery. In the case of intradomain allostery, the function and structure of one domain can be modulated through the ligand binding to allosteric sites of the domain itself.

sEH activity is allosterically regulated by an inhibitor. For example, the binding of an inhibitor to the N-phos active center changes the orientation of amino acids at the N–C domain interface [14]. In addition, ebselen covalently binds to the N-phos and inhibits the N-phos, which leads to an allosteric inhibition of the C-EH [5]. sEH activity is also allosterically regulated by its substrate. Positive cooperativity is found in the hydrolysis of EET by the C-EH [15].

Qiu et al. have reported that human sEH can be allosterically inhibited by the endogenous electrophilic lipid 15-deoxy-∆12,14-prostaglandin J2 and nitro fatty acids, 9- and 10-nitrooleate and 10-nitrolinoleate, via covalent adduction to the allosteric sites in the C-terminal domain [16]. Tran et al. have reported that lipid sulfates and sulfonates are allosteric competitive inhibitors of the N-phos activity of the mammalian soluble epoxide hydrolase. Their study also suggests that polyisoprenyl phosphates, substrates of the N-phos, inhibit the C-EH activity via a new binding site on the C-terminal domain [6].

Here, we describe an intradomain allostery in the sEH enzyme reciprocally regulated by the respective substrates for the C-EH and N-phos as effectors. We used single-domain enzymes corresponding to the N-phos (sEH-N) and C-EH (sEH-C) to elucidate the mechanism of allosteric regulation. The single-domain enzymes showed allostery, as did the full-length enzyme (sEH-FL), demonstrating intradomain cooperativity. The substrates, EET and LPA, allosterically affected both the N-phos and C-EH activities. sEH may link the metabolism of phospholipids and epoxy fatty acids for the control of inflammation and other relevant physiological functions through these mechanisms.

## 2. Results

### 2.1. Independent Intradomain Allostery in the N-phos and C-EH

**Figure 1:**
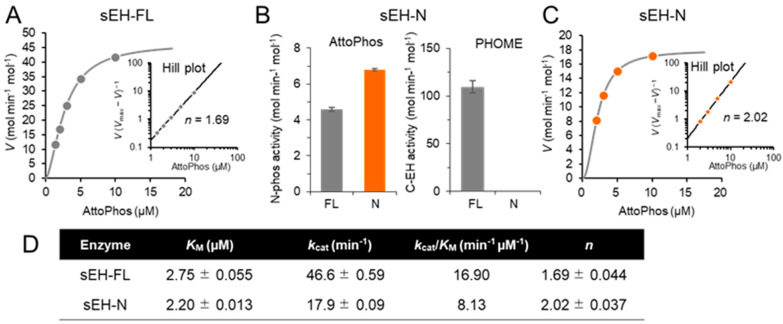
Intradomain allostery in N-phos activity. (**A**) Steady-state kinetics showing an allosteric effect of the N-phos substrate AttoPhos on the activity of sEH-FL. (**B**) Activity of the single-domain enzyme sEH-N when assayed at the substrate concentration of 5 μM. (**C**) Kinetic analysis of AttoPhos hydrolysis by sEH-N. (**D**) Summary of the kinetic parameters of N-phos activity in sEH-FL and sEH-N. In the [S]−*V* plots, each value represents the mean ± SD from triplicate determinations. In the Hill plots and kinetic data table, where regression data are shown, each value represents the mean ± SEM from triplicate determinations. Some error bars are not visible due to small errors.

**Figure 2:**
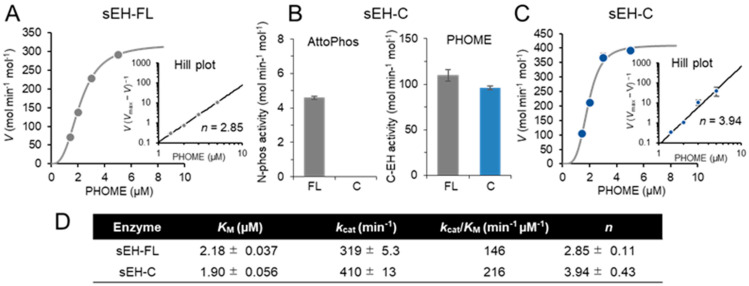
Intradomain allostery in C-EH activity. (**A**) Steady-state kinetics showing an allosteric effect of the synthetic C-EH substrate PHOME on the activity of sEH-FL. (**B**) Activity of the single-domain enzyme sEH-C when assayed at the substrate concentration of 5 μM. (**C**) Kinetic analysis of PHOME hydrolysis by sEH-C. (**D**) Summary of the kinetic parameters of C-EH activity in sEH-FL and sEH-C. In the [S]−*V* plots, each value represents the mean ± SD from triplicate determinations. In the Hill plots and kinetic data table, where regression data are shown, each value represents the mean ± SEM from triplicate determinations. Some error bars are not visible due to small errors.

When N-phos activity was determined using sEH-FL and the Attophos substrate, a positive cooperativity with a Hill coefficient (*n*) of 1.69 ± 0.04 was observed from steady-state enzyme kinetic analysis (Figure 1A). To elucidate the mechanism of the allosteric effect, we prepared the truncated enzyme sEH-N, which had no detectable C-EH activity (Figure 1B) but showed a positive cooperativity similar to that found in sEH-FL (*n* = 2.02 ± 0.04) (Figure 1C,D). Similar positive cooperativity (2.85 ± 0.11) was observed when C-EH activity was determined using sEH-FL and the PHOME substrate (Figure 2A). Again, the single-domain enzyme sEH-C, which had C-EH activity alone (Figure 2B), exhibited a positive cooperativity (*n* = 3.94 ± 0.43) (Figure 2C,D). Thus, both the N-phos and C-EH activities are regulated via intradomain allostericity via their respective substrates.

### 2.2. Allosteric Regulation of N-phos Activity by N-phos and C-EH Substrates

**Figure 3:**
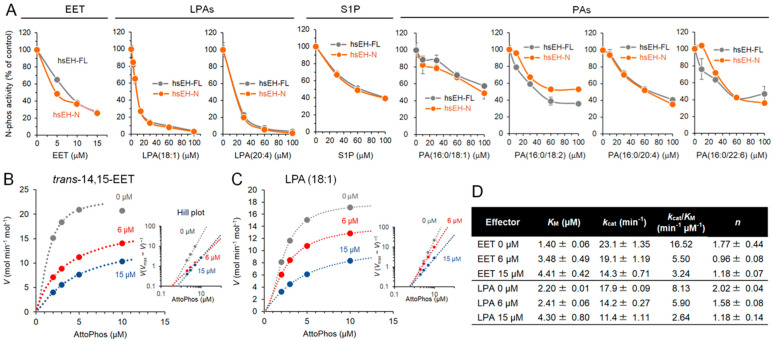
Allosteric regulation of N-phos activity by EET and LPA. (**A**) Effect of *trans*-14,15-EET, LPAs, and PAs on the N-phos activity of sEH-FL and sEH-N determined using the AttoPhos substrate. (**B**) Kinetic analysis of the effect of *trans*-14,15-EET on the AttoPhos hydrolysis by sEH-N. (**C**) Kinetic analysis of the effect of LPA (18:1) on the AttoPhos hydrolysis by sEH-N. (**D**) Summary of the kinetic parameters of N-phos activity in sEH-N. In the [S]−*V* plots, each value represents the mean ± SD from triplicate determinations. In the Hill plots and kinetic data table, where regression data are shown, each value represents the mean ± SEM from triplicate determinations. Some error bars are not visible due to small errors.

There is a structural similarity between LPA and EET in that both have a fatty acyl moiety. Therefore, we hypothesized that the substrates of the N-phos and C-EH might bind and affect each other’s allosteric sites, leading to a change in catalytic activity. We investigated the effect of the representative C-EH substrate *trans*-14,15-EET and the N-phos substrates LPA (18:1), LPA (20:4), and sphingosine 1 phosphate (S1P) on C-EH and N-phos activities. Interestingly, 14,15-EET attenuated the N-phos activities of sEH-FL and sEH-N to the same extent (Figure 3A). Since sEH-N has no detectable C-EH activity, this result unambiguously shows that 14,15-EET modulates N-phos activity via an intradomain mechanism. LPAs and S1P exhibited an inhibitory effect on the N-phos activity of sEH-FL and sEH-N to the same extent (Figure 3A). In contrast, phosphatidic acid (PA) has a structural moiety of LPA and is not an effective substrate for the N-phos [17] but acts as a modulator of N-phos activity. The potency of PAs with different acyl chains to inhibit N-phos activity was comparable to that of S1P (Figure 3A). We then performed kinetic analysis regarding the effect of EET and LPA (18:1) using sEH-N. As shown in Figure 3B, sEH-N-mediated AttoPhos hydrolysis exhibited a sigmoidal curve. The Hill plot showed a coefficient of 1.77 ± 0.44. The addition of 14,15-EET resulted in decreases in the *k*_cat_, from 23.1 ± 1.35 to 14.3 ± 0.71 min^−1^, and in the Hill coefficient, from 1.77 ± 0.44 to 1.18 ± 0.07, in the presence of 15 mM 14,15-EET. These data suggest that an intradomain allosteric mechanism may be involved in the decrease in the degree of positive cooperativity in sEH-N (see Section 3 for details). Kinetics analysis of the effect of LPA (18:1) on the N-phos activity of sEH-N showed a change in the *k*_cat_, from 17.9 ± 0.09 to 11.4 ± 1.11 min^−1^, and in the Hill coefficient, from 2.02 ± 0.04 to 1.18 ± 0.14, in the presence of 15 mM LPA (18:1) (Figure 3C,D). The fact that the *k*_cat_ value decreases with an increasing concentration of LPA (18:1) can exclude a typical competitive mechanism of inhibition. In addition, the decrease in the Hill coefficient to close to 1 shows a loss of the substrate (AttoPhos)-mediated positive cooperativity, suggesting an interaction of LPA (18:1) with the allosteric site that mediates the positive cooperativity (see Section 3 for a detailed interpretation). Thus, these data suggest that an allosteric mechanism predominates over the potential competition between AttoPhos and LPA (18:1), and an intradomain allosteric mechanism may be involved in the decrease in the degree of positive cooperativity in sEH-N.

### 2.3. Allosteric Regulation of the C-EH by N-phos and C-EH Substrates

**Figure 4:**
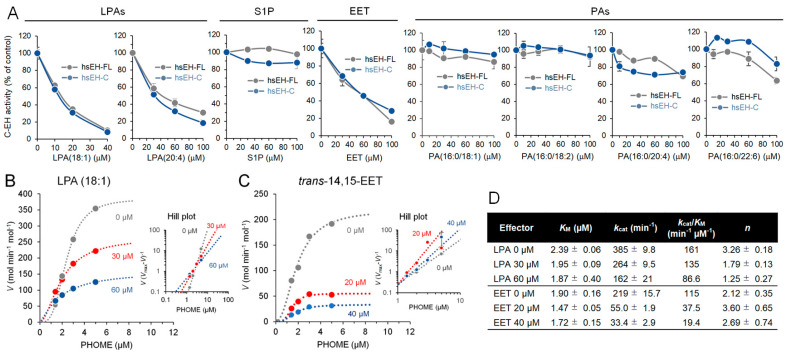
Allosteric regulation of C-EH activity by LPA and EET. (**A**) Effect of LPAs, *trans*-14,15-EET, and PAs on the C-EH activity of sEH-FL and sEH-C determined using the PHOME substrate. (**B**) Kinetic analysis of the effect of LPA (18:1) on the PHOME hydrolysis by sEH-C. (**C**) Kinetic analysis of the effect of *trans*-14,15-EET on the PHOME hydrolysis by sEH-C. (**D**) Summary of the kinetic parameters of C-EH activity in sEH-C. In the [S]−*V* plots, each value represents the mean ± SD from triplicate determinations. In the Hill plots and kinetic data table, where regression data are shown, each value represents the mean ± SEM from triplicate determinations. Some error bars are not visible due to small errors.

Next, we examined the effect of EET, LPAs, S1P, and PAs on the C-EH activity using sEH-FL and sEH-C. Similar to the results shown in Figure 3, hydrolysis of PHOME by sEH and sEH-C was inhibited by EET, LPAs, and S1P to the same extent, whereas PAs affected the activity minimally (Figure 4A). Kinetics analysis of LPA (18:1) action on sEH-C showed a dose-dependent reduction in the *k*_cat_, from 385 ± 10 to 162 ± 21 min^−1^, and in the Hill coefficient, from 3.26 ± 0.18 to 1.25 ± 0.27 in the presence of 60 μM LPA (18:1), suggesting an intradomain allosteric mechanism in the decrease in the degree of positive cooperativity in sEH-C (Figure 4B,D). Similarly, 14,15-EET decreased the *k*_cat_ from 219 ± 16 to 55.0 ± 1.9 and 33.4 ± 2.9 min^−1^ at 20 and 40 μM, respectively (Figure 4C,D). Thus, like the interpretation of the results of the sEH-N regulation by LPA described in Section 2.2, this result suggests an allosteric mechanism but not a simple competitive one in the 14,15-EET-mediated inhibition of sEH-C. On the other hand, 14,15-EET did not result in a decrease in the Hill coefficient, suggesting minimal impact on positive cooperativity (Figure 4C,D).

### 2.4. Prediction of Allosteric Sites by Molecular Docking Analyses

**Figure 5:**
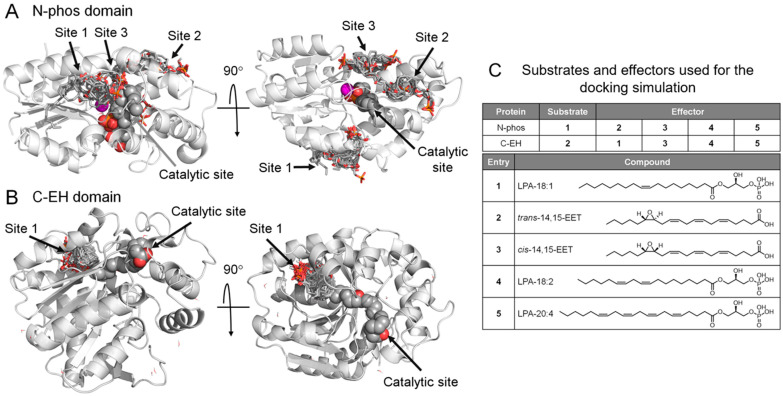
In silico prediction of potential allosteric sites in the N-phos and C-EH domains. (**A**) Superposition of all docking modes for the binding of test compounds **2**, **3**, **4**, and **5** to the N-phos monomer complexed with LPA (18:1) (**1**) at the catalytic site. (**B**) Superposition of all docking modes for the binding of test compounds **1**, **3**, **4**, and **5** to the C-EH monomer complexed with *trans*-14,15-EET (**2**) at the catalytic site. LPA (18:1) and *trans*-14,15-EET are represented as spheres in CPK color in panels (**A**) and (**B**), respectively. Test compounds are represented as tubes in CPK color. The Mg^2+^ ion is represented as a magenta-colored sphere. (**C**) Compounds tested for the docking simulations.

To assess the presence of a potential allosteric site that allows the binding of LPA and EET in each domain of sEH, we performed a docking analysis based on the known crystal structures of inhibitor-bound sEH. First, possible poses of the N-phos monomer and C-EH monomer were prepared from the available data [14,18], and the N-phos monomer/LPA (18:1) complex and the C-EH monomer/*trans*-14,15-EET complex, which can explain the catalytic mechanism, were predicted by the site docking analysis. The potential allosteric site for the test compound was predicted by blind docking analysis. As a result, three sites were predicted on the N-terminal domain (two of which, sites 2 and 3, are adjacent and may be occupied simultaneously by the ligand) (Figure 5A) and one on the C-EH (Figure 5B) as candidate sites for binding to LPA and EET (structure shown in Figure 5C).

## 3. Discussion

sEH is implicated in the regulation of diverse physiological and pathological processes, including inflammation. Therefore, sEH has drawn wide attention as a target of novel drug development [19,20,21,22,23]. However, no sEH C-EH-selective inhibitors have been approved worldwide. In addition, little is reported for the development of an N-phos inhibitor, possibly due to the limited information regarding its physiological role and interspecies differences in inhibitor sensitivity [13,14].

Insights into the allosteric regulation of sEH may provide a better opportunity for drug development via novel mechanisms. Kramer et al. have shown that sEH enzyme activity is allosterically regulated: upon binding of the N-phos inhibitor 3-(4-(3,4-dichlorophenyl)-5-phenyloxazol-2-yl) benzoic acid to the active site, the orientation of amino acids at the N–C interface of sEH changes [14]. Morisseau et al. also showed that ebselen covalently binds to the N-phos and inhibits the N-phos, which may trigger inhibition of the C-EH [8]. In the present study, we investigated the mechanism of allosteric regulation of sEH using sEH-FL and truncated enzymes with a single N-phos domain (sEH-N) and C-EH domain (sEH-C) and tested whether the allosteric nature of sEH is attributed to intradomain regulation or interdomain crosstalk between the C-EH and N-phos upon substrate binding. Since the substrates of the N-phos and C-EH are structurally similar with respect to the long-chain fatty acid moieties, we also tested whether the N-phos and C-EH are allosterically regulated by their natural substrates.

The results show that both the N-phos and C-EH activities are allosterically regulated by their respective substrates. As the positive cooperativity observed in sEH-FL is recapitulated by a single-domain enzyme, sEH-N and sEH-C, allostericity is concluded in each domain. Interestingly, the respective substrates reciprocally regulate the enzymatic activity of sEH-N and sEH-C: the N-phos and C-EH are regulated by EET and LPA. Again, these effects are concluded in each of the domains. The kinetic analysis data are consistent with the docking simulation results, as described below.

Docking simulations predicted the presence of allosteric sites in sEH-N and sEH-C that allow the binding of EET and LPA (Figure 5). Three potential allosteric sites are predicted in the sEH-N/LPA (18:1) complex, and one allosteric site for the C-EH monomer/*trans*-14,15-EET complex. All of these allosteric sites are predicted to bind both 14,15-EET and LPAs. These results support the intradomain allostery suggested by the enzyme kinetic analyses. In sEH-N, LPA may bind to at least one of the possible sites in addition to binding to the catalytic site, exhibiting positive cooperativity. At a higher concentration, LPA may bind to an additional allosteric site exhibiting allosteric inhibition. The fact that the second allosteric site binding is accompanied by a decrease in the Hill coefficient (Figure 4B) suggests that this binding may affect the conformation in the primary allosteric site to diminish positive cooperativity. The kinetic data for the 14,15-EET-mediated sEH-N inhibition are similar to those for the LPA inhibition, and it is likely that the mechanism involved in the 14,15-EET action is similar to that for LPA. In sEH-C, the positive cooperativity may be derived from substrate binding to the predicted allosteric site in addition to the catalytic site. Unlike the case of the LPA effect on sEH-N, positive cooperativity is not diminished by higher concentrations of 14,15-EET. This result is consistent with the docking prediction of one allosteric site. On the other hand, the LPA effect on sEH-C is accompanied by a decline in positive cooperativity, as evidenced by a decrease in the Hill coefficient. Taking the one-allosteric site model for sEH-C, it may be possible that LPA competes with 14,15-EET to bind to the allosteric site, and this LPA binding does not result in positive cooperativity, diminishing 14,15-EET-mediated cooperativity.

This in silico study was performed as an initial step to understand the details of allosteric regulation of sEH, and we took an approach to roughly estimate the location of potential sites. This was because the biochemical data suggested complex mechanisms for the allosteric regulation by substrates. Further investigation is required to elaborate on the structural basis of the allosteric regulation.

Taken together, these results show that sEH-N and sEH-C are independently regulated via an intradomain allostery mediated by a substrate of its own and by that of the other domain. This mechanism may allow sEH to precisely control the metabolism of anti-inflammatory epoxy fatty acids and the signaling lipid LPA, depending on the levels of these metabolites. Thus, sEH may link the metabolism of phospholipids and epoxy fatty acids for the control of inflammation and other relevant physiological functions through mutual substrate-mediated allosteric regulation. In addition, this mechanism may explain why sEH has evolved to have two different enzyme activities in one molecule.

## 4. Materials and Methods

### 4.1. Materials

Recombinant human sEH (N-terminal His-tagged protein; item No. 10011669) was purchased from Cayman Chemical (Ann Arbor, MI, USA). The N-phos substrate AttoPhos was obtained from Promega (Fitchburg, WI, USA), and the C-EH substrate (3-phenyl-oxiranyl)-acetic acid cyano-(6-methoxy-naphthalen-2-yl)-methyl ester (PHOME) was obtained from Cayman Chemical (Ann Arbor, MI, USA). All the compounds tested were purchased from Cayman Chemicals: 14,15-epoxyeicosatrienoic acid (14,15-EET), lysophosphatidic acids; LPA (18:1), LPA (20:4), phosphatidic acid (PA) with different fatty acyl chains, PA (16:0/18:1), PA (16:0/18:2), PA (16:0/20:4), and PA (16:0/22:6). Unless otherwise indicated, all the compounds were assayed as DMSO solutions at the final DMSO concentrations of 1% (*v*/*v*) or less.

### 4.2. Preparation of Single-Domain Proteins of sEH

Recombinant enzymes of human sEH C-EH domain (sEH-C) and N-phos domain (sEH-N) were prepared in Protein Express Co. (Chiba, Japan) as follows: The plasmids sEH-H-aa222-555-C-His-pET-28b(+) and sEH-P-aa2-224-pET29BH4 [14] were kindly provided by Ewgenij Proschak (Goethe University Frankfurt, Frankfurt, Germany). BL21(DE3) Competent Cells (Novagen, WI, USA) were transformed using sEH-P-aa2-224-pET29BH4 and sEH-H-aa222-555-C-His-pET-28b(+). Transformants were cultured in 50 mL TB medium (47.6 g/L Terrific Broth (Invitrogen, Waltham, MA, USA) and 0.4% Glycerol) at 37 °C for 18 h. The main culture was performed in 1.2 L scale TB medium (120 mL × 10 bottles) at 37 °C, 120 rpm, for 3 h, followed by induction of the target protein with a final concentration of 0.5 mM IPTG. Induction conditions were 21 °C for 18 h for sEH-N and 16 °C for 22 h for sEH-C. After completion of incubation, the collected bacteria were incubated in 120 mL of binding buffer (sEH-N: 25 mM Bis-Tris, 500 mM NaCl, pH 7.0 in cOmplete™, EDTA-free, Roche, Basel, Switzerland; sEH-C: 50 mM Tris-HCl pH 8, 500 mM NaCl in cOmplete™, EDTA-free, Roche), sonicated, and centrifuged at 20,000× *g* for 60 min to obtain supernatant. After filtration, the sample was applied to a 5 mL Ni-NTA Cartridge (FUJIFILM Wako Pure Chemicals, Osaka, Japan) pre-equilibrated with a binding buffer. The column was washed with a binding buffer, and the bound protein was eluted with a gradient of imidazole (0 mM to 500 mM, 5 mL × 20 fractions) in a binding buffer. Each fraction was analyzed using SDS-PAGE and Western blotting. Fractions containing the target protein were collected, and the buffer was changed by dialysis to 50 mM acetate and 10 mM MgCl_2_, pH 5.75, for sEH-N and 50 mM NaCl, 50 mM sodium phosphate, 10% (*v*/*v*) glycerol, and 2 mM DTT, pH 7.4, for sEH-C. The protein concentration was determined by the Bradford assay.

### 4.3. Assay for the N-phos

To a 96-well black plate, 60 µL of buffer A (25 mM Bis-Tris-HCl, pH 7.0, 1 mM MgCl_2_, and 0.1 mg mL^−1^ bovine serum albumin) containing 60 ng of sEH and 20 µL of effector solution of any concentration diluted with buffer A were added. Subsequently, 20 µL of 25 µM AttoPhos in buffer A was added. The reaction was stopped by adding 50 µL of 10 mM NaOH after incubation at 37 °C for 90 min. Fluorescence intensity at excitation and emission wavelengths of 450 nm and 545 nm, respectively, was measured. For the kinetic analysis, human N-phos activity was measured using AttoPhos and sEH-N in the presence of 0, 6, and 15 µM of 14,15-EET and LPA (18:1).

### 4.4. Assay for the C-EH

The C-EH activity was determined using PHOME as a substrate. To a 96-well black plate, 60 µL of buffer A containing 60 ng of sEH enzymes and 20 µL of effector solution of any concentration diluted with buffer A were added. The reaction was started by adding 20 µL of 25 µM PHOME in buffer A. Fluorescence intensity at 465 nm (with excitation at 330 nm) was measured kinetically at 37 °C for 30 min.

### 4.5. Data Analysis

Enzyme activity is expressed as a mol of substrate hydrolyzed per min per mol of enzyme (mol min^−1^ mol^−1^). For the kinetic analysis, the concentration of PHOME and AttoPhos was changed, as shown in each figure. Data were analyzed using SigmaPlot 14.5 (Inpixon, Palo Alto, CA, USA). Since all the data obtained appeared to fit the Hill equation, the kinetic parameters *K*_M_, *V*_max_, *k*_cat_, and the Hill coefficient (*n*) were determined by regression analysis based on the Hill equation.

### 4.6. In Silico Prediction of Allosteric Sites

To perform docking simulations using the AutoDock Vina version 1.1.2 [24], the planar structure of test compounds (see Figure 5C) was three-dimensionalized using Balloon version 1.8.1 [25,26] under an MMFF94 force field. The compounds’ protonation state was prepared using Babel version 2.4.1 [27] under the physiological condition (pH 7.4). Simultaneously, Gasteiger charges were assigned and converted to the pdbqt format.

The X-ray crystal structure (PDB ID: 6I5E A chain; resolution 2.60 Å) [18] for the C-EH monomer was refined using Homology Modeling Professional for HyperChem software, version 8.1.5 (HMHC) [28]. After adding hydrogen atoms automatically, aspartic and glutamic acid residues were treated as anions, and lysine, arginine, and histidine residues were treated as cations under physiological conditions. The N- and C-terminal residues were treated as zwitterions. Structural refinement was performed under the AMBER99 force field using the following parameters: root-mean-square gradient, 1.0 kcal·mol^−1^·Å^−1^; algorithm, Polak–Ribière; cut-off, switched (outer radius = 14.0 and inner radius = 10.0); 1–4 van der Waals scale factor, 0.5; 1–4 electrostatic scale factor, 0.833; dielectric scale factor, 1.0; and distance-dependent dielectric condition. Subsequently, partial optimization for all components, excluding heavy atoms of the main chain and crystal waters, was performed. The obtained structure was converted to the pdbqt format using AutoDockTools version 1.5.7.

The X-ray crystal structure (PDB ID: 5MWA; resolution 1.55 Å) [14] for the N-phos monomer was refined using HMHC. Missing parts (Cys74−Lys84 and Ala131−Gln138) were modeled using a template (PDB ID: 5ALU) [29]. After assigning semi-empirical MNDO/d Mulliken atomic charges for the complexed inhibitor (8SQ) and +2 formal charge for the Mg ion in the active site, structural refinement was performed by the same method as described for the C-EH monomer. To optimize the missing parts, distance restraint conditions (7.0 kcal·mol^−1^·Å^−2^) were applied to all the heavy atoms, excluding the missing parts, of the prepared structures. Then, the structure was subjected to low-temperature molecular dynamics annealing (starting temperature, 0 K; heat time, 30 ps; simulation temperature, 300 K; run time, 100 ps; final temperature, 0 K; cooling time, 30 ps; step size, 0.001 ps; and temperature step, 0.01 K). Finally, all distance restraint conditions were removed, and the structure was further optimized to obtain the final structure. The precision of the final structures was confirmed using the Ramachandran plot program of HMHC. The complexed inhibitor (8SQ) and all the crystal waters, excluding the waters related to chelation with the Mg^2+^ ion in the active site, were removed. The obtained structure was converted to the pdbqt format using AutoDockTools.

The site docking simulations for the C-EH monomer with *trans*-14,15-EET were performed using AutoDock Vina. Using the coordinates of the complexed inhibitor (1LF in PDB ID: 4JNC) [30], a configuration file with cavity information (the value of the grid box was set to center x = −65.675 Å, center y = −20.389 Å, and center z = −15.676 Å with size x = 20.015 Å, size y = 22.954 Å, and size z = 25.554 Å) was prepared using Docking Study with HyperChem software, version 8.1.5 (DSHC) [31], and other docking conditions were set to the following values. The top nine docking modes per trial compound were maximally output, and the exhaustiveness value was set to 100. Consequently, the reliable C-EH monomer/*trans*-14,15-EET complex was obtained. That is, the oxygen atom of the epoxy moiety of *trans*-14,15-EET interacted with the epoxy positioners (Tyr383 and Tyr466), and, on the opposite side, the hydrogen atom at the 14 position of *trans*-14,15-EET interacted with the catalytic triad (Asp335, Asp496, and His524).

The site docking simulations for the N-phos monomer with LPA (18:1) were performed using AutoDock Vina. Using the coordinates of the complexed inhibitor (8SQ), a configuration file with cavity information (the value of the grid box was set to center x = −9.207 Å, center y = −10.491 Å, center z = 16.669 Å with size x = 25.647 Å, size y = 23.561 Å, and size z = 22.136 Å) was prepared using DSHC, and other docking conditions were set to the above-mentioned values. As a result, the reliable N-phos monomer/LPA (18:1) complex was obtained. The phosphoric moiety of LPA (18:1) interacted with the Mg^2+^ ion, Asn124, and Asn189, and the hydrophobic side chain of LPA (18:1) interacted with Val19, Phe20, Phe41, Trp63, Phe92, and Ile96.

These stable docking structures of *trans*-14,15-EET and LPA (18:1) were further optimized in their cavities under the AMBER99 force field.

For the C-EH monomer/*trans*-14,15-EET and N-phos monomer/LPA (18:1) complexes, simulations of blind docking of test compounds were performed using AutoDock Vina. For the C-EH monomer/*trans*-14,15-EET complex, a configuration file with cavity information (the value of the grid box was set to center x = −60.563 Å, center y = −14.345 Å, and center z = −17.945 Å with size x = 58.979 Å, size y = 53.998 Å, and size z = 55.485 Å) was prepared using DSHC, and other docking conditions were set to the above-mentioned values. For the N-phos monomer/LPA (18:1) complex, a configuration file with cavity information (the value of the grid box was set to center x = −15.379 Å, center y = −6.660 Å, and center z = 17.165 Å with size x = 51.690 Å, size y = 57.075 Å, and size z = 59.007 Å) was prepared using DSHC, and other docking conditions were set to the above-mentioned values.

## Data Availability

The data presented in this study are available in this article.
